# Influence of Content and Type of Lanthanide on the Structure of Ln_2_O_3_-Covered Carbon Nanoflakes: The EPR and XPS Study

**DOI:** 10.3390/nano15131016

**Published:** 2025-07-01

**Authors:** Serguei V. Savilov, Evgeniya V. Suslova, Alexander N. Ulyanov, Konstantin I. Maslakov, Sergey V. Maximov, Denis A. Shashurin, Georgy A. Chelkov

**Affiliations:** 1 Chemistry Department, Lomonosov Moscow State University, Moscow 119991, Russia; 2 Faculty of Medicine, Medical Scientific-Educational Institute, Lomonosov Moscow State University, Moscow 119991, Russia; 3 Joint Institute for Nuclear Research, Dubna 141980, Russia

**Keywords:** lanthanides, gadolinium, carbon nanoflakes, electronic structure, electron paramagnetic resonance, X-ray photoelectron spectroscopy

## Abstract

Synthesized Ln_2_O_3_ (Ln = La, Nd or Gd) nanoparticles with sizes of 1–3 nm, 5–6 nm and 10–15 nm were stabilized by carbon nanoflakes (CNFs). The weight content of Ln_2_O_3_ in the Ln_2_O_3_/CNF composites was 20–50 wt. %, which makes these composites potentially suitable for practical use as computed tomography and magnetic resonance imaging contrast agents. The structure of CNFs and Ln_2_O_3_/CNF composites was investigated by X-ray diffraction data, X-ray photoelectron spectroscopy (XPS) and electron paramagnetic resonance (EPR). The EPR spectra of raw CNFs were silent. The oxidation of the CNF surface resulted in the appearance of paramagnetic centers associated with two types of unpaired electrons in the carbon support. After impregnation of the CNFs with the Ln^3+^ ion solution, the number of unpaired electrons was reduced, presumably due to the formation of C–O–Ln bonds. All Ln^3+^ ions changed the composites’ EPR spectra by reducing the number of unpaired electrons in the CNF structure.

## 1. Introduction

Carbon nanomaterial (CNM) composites containing the rare-earth oxides (Ln_2_O_3_) have been widely applied as functional materials. La_2_O_3_/graphene oxide (GO) is used as a catalyst in the synthesis of dimethyl carbonate [1]. La_2_O_3_/reduced GO and La_2_O_3_/carbon nanotubes (CNTs) can be used as electrode materials for asymmetric supercapacitors [2,3]. GO-supported C@Nd_2_O_3_ is a photocatalyst [4]. Carbon-supported rare-earth oxides are used to remove antibiotics [5]. Gd_2_O_3_/CNT composites [6], Gd_2_O_3_/graphene quantum dots [7], Gd_2_O_3_/GO [8], endohedral fullerene Gd@C_60_ [9], Gd_2_O_3_ stabilized by carbon nanoflakes (CNFs) and core shell (Gd_2_O_3_/CNFs)@C particles [10] are described as contrast agents for magnetic resonance imaging (MRI). The CNFs covered with *3d* and *4f* metals are effective contrast agents for photon-counting computed tomography [11,12,13,14].

Ln_2_O_3_/CNMs can be synthesized by several methods such as the high-energy ball milling of CNMs and lanthanide compounds [15], the mixing of GO and Nd_2_O_3_ [4], the interaction of CNMs with lanthanide metals in molten chlorides [16], the intercalation of CNMs with LnCl_3_ vapors [17] or the impregnation of CNMs with Ln-containing precursors [11,12,13,14,18,19,20]. The oxidation of the carbon surface results in the formation of carboxyl and hydroxyl surface groups that enhance the uniform sorption and distribution of Ln^3+^ ions [18,19]. However, questions about how Ln^3+^ ions bind with the carbon matrix remain open. It can be assumed that CNM dimensions can affect the content of adsorbed Ln^3+^. In [19], the maximum amount of Gd^3+^ adsorbed onto the surface of 0D nanodiamond (ND) particles was limited to below 3.5 wt. % even at higher Gd^3+^ concentrations in the solution because of Gd(OH)_3_ precipitation. In [13], the authors showed that the increase in La^3+^ concentration in the water solution increased the size of La_2_O_3_ particles deposited on the 2D CNFs.

The way Ln^3+^ ions bind to the carbon surface is critically important for some potential application of Ln_2_O_3_/CNMs, for example, their use as contrast agents for MRI and computed tomography in medical diagnostics and biomedical studies. The stable bonding of Ln^3+^ ions to the CNM particles ensures their stability in biological media and, subsequently, their biocompatibility and safety. However, for practical applications, these particles should be insoluble and contain sufficient Ln content for its determination by MRI and computed tomography [21]. We demonstrated early that Ln_2_O_3_/CNM (Ln = La, Nd, Gd) composites could be considered effective contrast agents when the content of Ln in the composition is at a minimum of 15–20 wt. % [10,11,12,13,14].

The nature of Ln^3+^ interactions with the CNM surface has been investigated using theoretical studies [22], transmission electronic microscopy (TEM) [18], IR spectroscopy, X-ray photoelectron spectroscopy (XPS) [23] and electronic paramagnetic resonance (EPR) [19,24,25,26]. Specifically, the EPR spectra are described for numerous CNMs [27]. It was shown that they are very sensitive to the chemical bonded and adsorbed oxygen in the CNMs [28,29,30]. Gd^3+^ ions are suitable for EPR examination both in ordered and disordered matrices, providing information about oxygen vacancies and the coordination environment of Ln^3+^ [31]. EPR spectra combined with ^13^C NMR and DFT have enabled estimations of metal–surface distances in Cu-, Co- and Gd-grafted NDs, Fe-grafted graphenes and Mn-grafted GO [19,25]. However, these works predominantly studied metal–carbon composites with a metal content that was lower than what is typically seen in most biomedical studies.

In the present study, we used EPR and XPS to assess Ln_2_O_3_ (Ln = La, Nd or Gd) deposited on the CNF composites, which can be used as potential contrast agents for photon-counting computed tomography [12,13,14]. The aim of this work is to investigate how the nature and content of Ln_2_O_3_ affect the EPR spectra of Ln_2_O_3_/CNF composites to determine how Ln^3+^ is bound to the carbon matrix.

## 2. Experiment

### 2.1. Synthesis of Carbon Nanoflakes and Ln_2_O_3_/CNF Composites

CNFs were synthesized by the pyrolysis of hexane (99.8%, Reachim, Penza, Russia) at 900 °C in the presence of a MgO template [32]. The MgO template was removed by dissolution in boiling HCl (99.9% Reachim, Penza, Russia). The CNF surface was oxidized by refluxing with 67% HNO_3_ (99.999%, Chimmed, Moscow, Russia) for 1 h, followed by washing with distilled water and drying at 110 °C for 10 h. The oxidized CNFs were denoted as CNFs_ox. CNFs_ox were treated at 400 °C for 30 min under N_2_ gas to obtain a reference defunctionalized sample named CNFs_ox_400.

The synthesis of Ln_2_O_3_ (Ln = La, Nd or Gd) nanoparticles stabilized with a CNF matrix (Ln_2_O_3_/CNFs) has been described in detail in our previous studies [12,13]. The Ln_2_O_3_/CNF samples were prepared by the impregnation of CNFs_ox with Ln(NO_3_)_3_·6H_2_O (99%, China Northern Rare Earth Group High-Tech Co., Ltd., Baotou, China) solution in ethanol (99.99%, Merck, Darmstadt, Germany). The solvent was further evaporated, and nitrate was decomposed at 400 °C under nitrogen (99.999%, Logika Ltd., Moscow, Russia) flow. The calculated mass content of Ln_2_O_3_ was 20, 30, 40, and 50 wt. %.

### 2.2. Methods of Investigation

The specific surface area (S_BET_) was determined by low-temperature nitrogen physisorption on an Autosorb-1C/QMS (Quantachrome Inc., Boynton Beach, FL, USA) analyzer. Prior to the measurement, the samples were degassed in a vacuum at 300 °C for 3 h.

High-resolution transmission electron microscopy (HRTEM) images were recorded on a JEOL 2100 F/Cs (Jeol, Tokyo, Japan) microscope operated at 200 kV and equipped with a UHR pole tip as well as a spherical aberration corrector (CEOS, Heidelberg, Germany) and an EEL spectrometer (Gatan, Munich, Germany). The morphology of the samples was characterized using a JEOL JSM-6390LA scanning electron microscope (Jeol, Tokyo, Japan) operating at 25 kV.

X-ray photoelectron spectroscopy (XPS) was performed on an Axis Ultra DLD spectrometer (Kratos Analytical, Milton Keynes, UK) with a monochromatic Al Kα source (*hν* = 1486.7 eV) operated at 150 W. Survey XPS spectra were recorded with an analyzer pass energy of 160 eV and 1 eV steps. High-resolution spectra were acquired at a pass energy of 40 eV and a step size of 0.1 eV.

X-ray diffraction patterns were recorded in the 2θ range of 2–80° using the Stadi-P (Stoe & Cie, Darmstadt, Germany) instrument equipped with a CuKα (1.54060 Å) radiation source.

Electron paramagnetic resonance (EPR) data were collected with a BRUKER EMX 6/1 spectrometer (Karlsruhe, Germany) at 9.8–9.9 GHz (X-band) at room temperature. The background signal was subtracted using the Bruker WinEPR System Version 2.11b software. The reference sample was TEMPO (2,2,6,6-tetramethyl-1-piperidinyloxyl) in toluene solution with the number of paramagnetic centers N_st_ = 2.0792·10^16^ spins. The spin number N of the investigated samples was calculated according to the following equation:

(1) N = (Q_st_/Q)·(DI∙N_st_)/DI_st_,

where Q_st_ and Q are the quality factors of the resonator with the reference and studied samples, respectively, and DI and DI_st_ are the double integrated intensities of the resonance spectrum lines of the studied and reference samples, respectively. The DI values were derived from the integration of the initial raw experimental spectra with the consequent normalization to the sample mass. The Q/Q_st_ ratio was 0.9.

## 3. Results

### 3.1. Physicochemical Properties of Carbon Matrix

**Figure 1:**
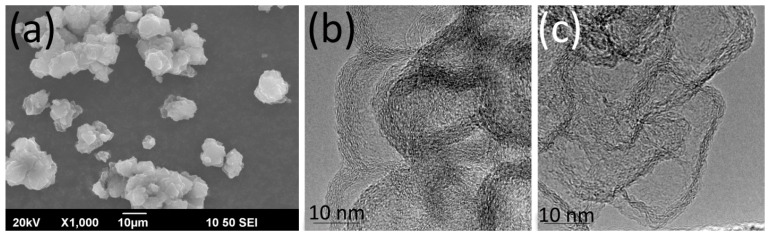
SEM (**a**) and TEM (**b**,**c**) images of CNFs (**a**,**b**) and oxidized CNFs_ox (**c**).

The morphology of CNFs replicated the shape of the MgO template particles and looked like stacked plates (Figure 1a). The analysis revealed that individual CNF particles comprised 7–15 graphene layers (Figure 1b). Following oxidative treatment, the number of carbon layers in the particles decreased (Figure 1c) because of the degradation of the upper layers [33]. This surface oxidation of CNFs was essential to ensure the uniform distribution of Ln^3+^ ions during impregnation with the Ln(NO_3_)_3_·6H_2_O solutions [18].

The S_BET_ values of CNFs_ox and CNFs_ox_400 were 401 and 374 m^2^·g^−1^, respectively.

**Figure 2:**
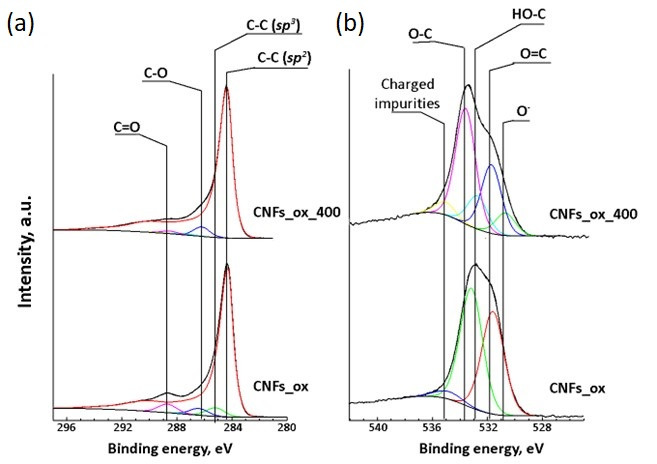
Curve-fitted C1s (**a**) and O1s (**b**) XPS spectra of CNFs_ox and CNFs_ox_400.

Fitting of the C1s XPS spectra of CNFs_ox and CNFs_ox_400 revealed several components with different binding energies attributed to different carbon species: specifically, to *sp*^2^- (at 284.4 eV), *sp*^3^-hybridized C atoms (285.1 eV) and different oxygen-bonded carbon atoms in C–O (286.2 eV), C=O (287.4 eV) and COOH (288.6 eV) functional groups (Figure 2a). The O1s XPS spectra mainly confirmed the presence of oxygen atoms attributed to aromatic O=C (531.6 eV), hydroxyl C–OH and aliphatic O=C (532.6 eV) species, and single-bonded oxygen in carboxylic groups (533.5 eV) (Figure 2b). The total oxygen content in the CNFs, oxidized CNFs_ox and defunctionalized CNFs_ox_400 samples was 1.9, 11.1 and 6.5 at. %, respectively.

**Figure 3:**
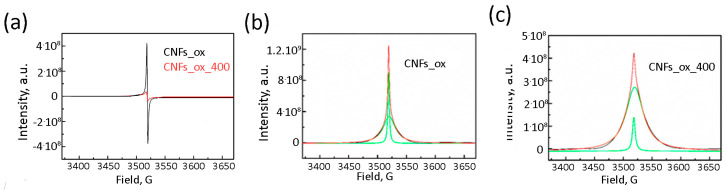
EPR spectra of CNFs_ox (black) and CNFs_ox_400 (red) (**a**). Absorption EPR spectra of CNFs_ox (**b**) and CNFs_ox_400 (**c**). Green lines represent Lorentzian components best fitted to the experimental spectra (**b**,**c**).

The EPR spectra of raw CNFs were silent, while the EPR and absorption EPR spectra of CNFs_ox and CNFs_ox_400 show a strong paramagnetic response (Figure 3). The absorption spectra of all samples can be well fitted with two Lorentzian lines with different intensities (DI), widths (Δ*H*) and positions (*g*-factor). The DI values were obtained by the double integration of the raw experimental spectra with consequent normalization to the sample mass. All numerical values are shown in Table 1.

### 3.2. Physicochemical Properties of Ln_2_O_3_/CNF Composites

**Figure 4:**
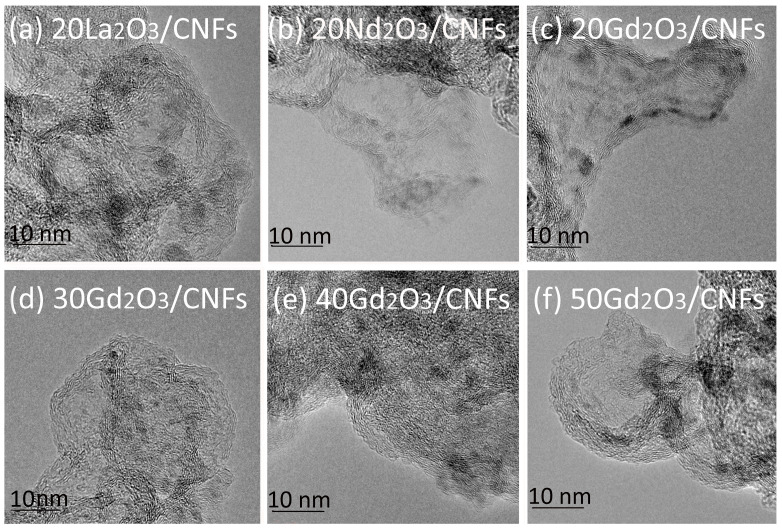
TEM images of Ln_2_O_3_/CNFs (Ln = La, Nd or Gd) with Ln_2_O_3_ weight content of 20% (**a**–**c**), 30% (**d**), 40% (**e**) and 50% (**f**).

**Figure 5:**
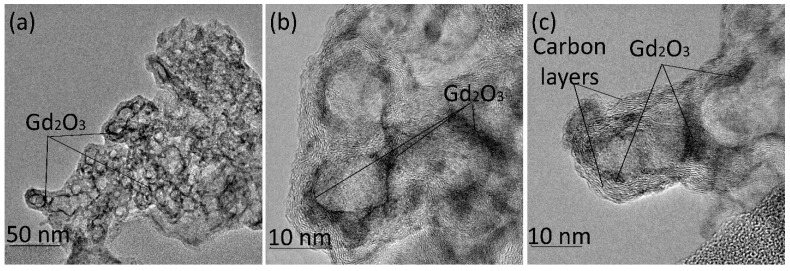
Gd_2_O_3_ localization on the CNF surface in 50Gd_2_O_3_/CNFs ((**a**) - overview; (**b**,**c**) - detailed TEM images).

According to the TEM images, the Ln_2_O_3_ particles were uniformly distributed over the CNF surface (Figure 4). The TEM images demonstrated that the Ln_2_O_3_ particle size increased with an increase in the Ln_2_O_3_ mass content from 20 up to 50 wt. %. For example, the size of the Gd_2_O_3_ particles grew from 2–3 nm up to 15–20 nm with an increase in Gd_2_O_3_ weight from 20 up to 50 wt. % (Figure 4c–f and Figure S1). This fact agrees with the previous study [13]. Ln_2_O_3_ particles with a high Ln_2_O_3_/CNF content lost their spherical shape (Figure 5a) and located mainly along the inner perimeter of the CNFs (Figure 5b,c). This is in agreement with the data on the maximum surface energy at the edge and corners of carbon nanoparticles [34].

**Figure 6:**
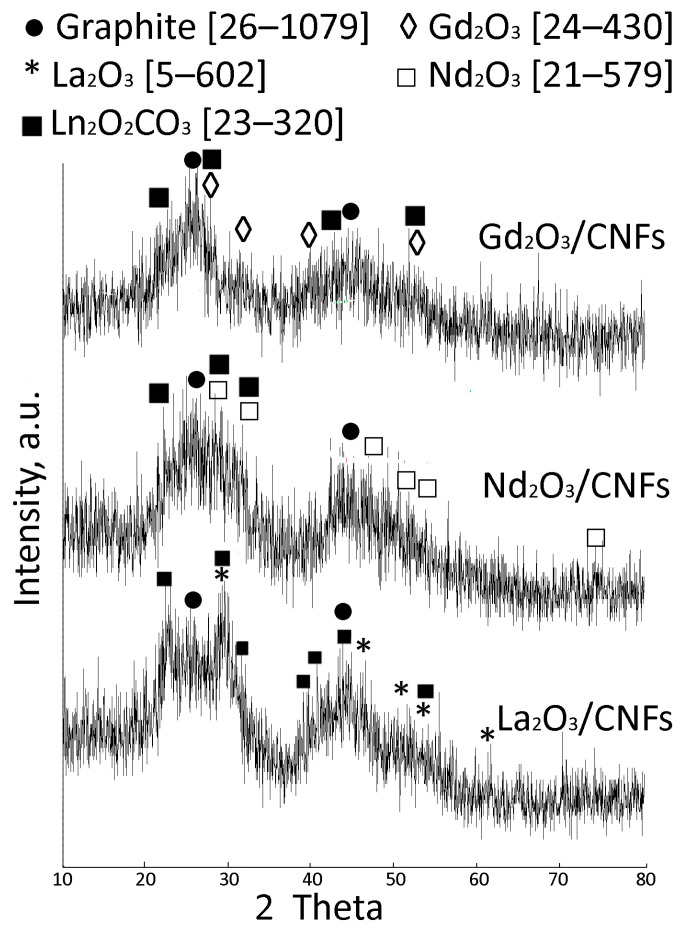
XRD patterns of 20Ln_2_O_3_/CNFs.

Figure 6 shows the XRD patterns of the samples. The carbon phase included reflexes at *2θ* ~ 25 and 44.6 (card (26–1079)). The nanosize of the Ln-containing particles resulted in low intensive reflexes close to the Ln_2_O_3_ and/or Ln_2_O_2_CO_3_ phases. Both Ln_2_O_3_ and Ln_2_O_2_CO_3_ are insoluble and do not produce free Ln^3+^.

**Figure 7:**
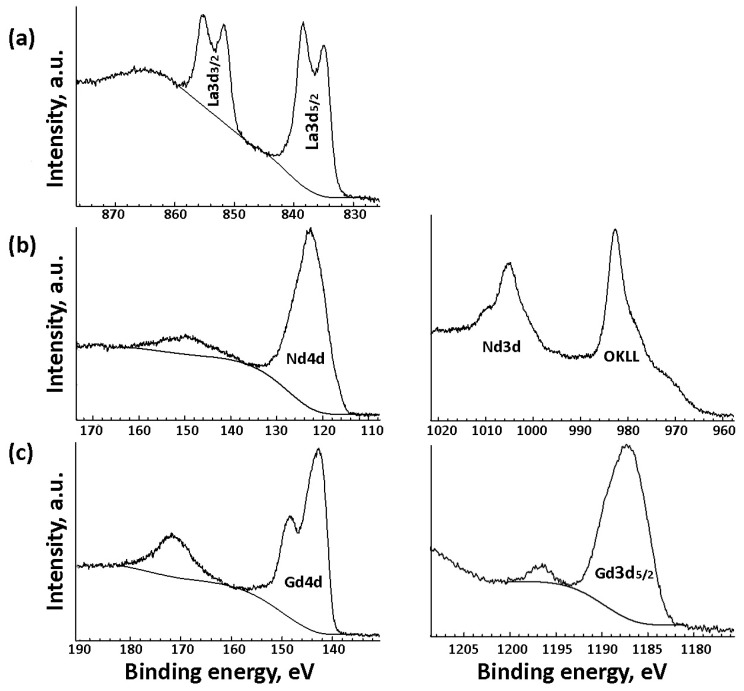
La3d (**a**), Nd4d, Nd3d (**b**), Gd4d and Gd3d (**c**) XPS spectra of 20Ln_2_O_3_/CNF (Ln = La, Nd or Gd) samples.

After Ln^3+^ deposition on the CNFs_ox surface followed by annealing at 400 °C, the XPS spectra of the Ln_2_O_3_/CNF samples showed a La3d_5/2_ peak at a binding energy of 834.9 eV, a Nd3d_5/2_ peak at 982.6 eV and a Nd4d peak at 122.7 eV, a Gd3d_5/2_ peak at 1187.2 eV and a Gd4d peak at 142.8 eV (Figure 7). These binding energies and the presence of intense shake-up satellites are characteristic of Ln^3+^ ions both in Ln_2_O_3_ and other compositions [15,35]. The separation of 3.5 eV between the main La3d_5/2_ peak and the satellite in the spectrum of La_2_O_3_/CNFs (Figure 7a) is close to that in La_2_O_2_CO_3_ [36].

**Figure 8:**
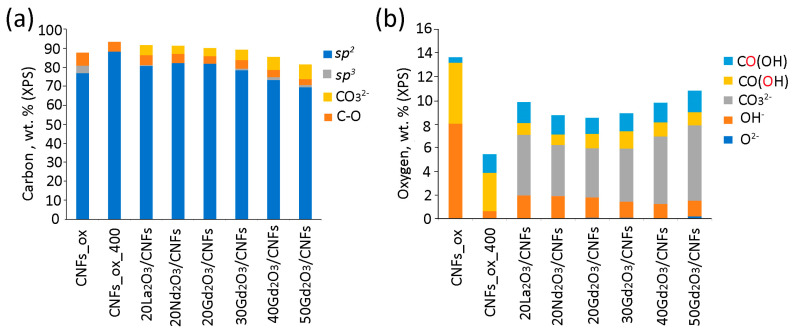
Ratio between different carbon (**a**) and oxygen (**b**) atom types according to XPS.

The fitting of the C1s XPS spectra revealed components of *sp*^2^- (at 284.4 eV) and *sp*^3^-hybridized (285.1 eV) carbon species and oxygen-bonded carbon atoms in C–OH (286.2 eV), C=O (287.4 eV) and COOH (288.6 eV) species (Figure S2). A component with a binding energy of 289.3 eV in the C1s spectra confirms the presence of carbonates (Figure S2a). The O1s XPS spectra show the components of lattice oxygen (O^2−^) in oxides (at 528.9 eV), aromatic O=C and CO_3_^2−^ (531.6 eV), HO–C (533.2 eV), and O=C (532.6 eV) and OH–C (533.5 eV) species in carboxylic groups (Figure S2b). Thus, Ln^3+^ ions in the samples were predominantly coordinated with lattice oxygen and carbonate groups. The ratios of different types of C and O species, calculated from the C1s and O1s high-resolution XPS spectra, are presented in Figure 8.

The EPR and absorption EPR spectra of xLn_2_O_3_/CNFs (Ln = La, Nd or Gd; x = 30, 40 or 50) are shown in Figure S3. The parameters of the EPR spectra are summarized in Table 1.

## 4. Discussion

### 4.1. The Effect of Carbon Nanoflakes on the EPR Spectra

**Figure 9:**
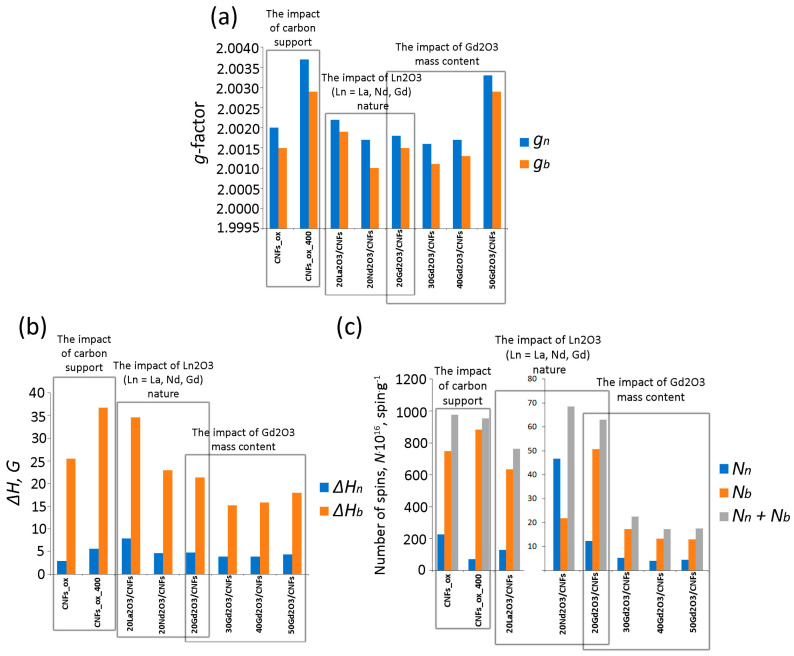
The changes in *g*-factor (**a**), linewidth (Δ*H*) (**b**) and spin number (*N*) (**c**) calculated from EPR spectra of samples. Subscripts *n* and *b* denote narrow and broad components of the spectra. The uncertainties are 3% for Δ*H*, 0.0000(5) for g-factors and less than 15% for spin numbers.

Previous studies established that CNFs have no EPR signal [37]. The EPR response could be registered only after CNF oxidation resulted in the appearance of the surface oxygen-containing groups that increase the defectiveness of GNFs, introduce *sp*^3^-hybridized carbon atoms (Figure 3) and decrease the particle size (Figure 1c), thereby enhancing interlayer interactions [38]. The EPR spectra of CNFs_ox demonstrate a superposition of a broad and a narrow line that correspond to two different types of paramagnetic centers with *g* factors of 2.0015 and 2.0037 (Figure 9a), which is typical for nanosized carbon. Two types of paramagnetic centers and *g* factors were previously described for GO [39,40], CNTs [37,41] and 5 nm NDs, while the bulk diamond [42] and carbon nanodots [43] show only one type of paramagnetic center. The temperature dependence of the EPR signal of GO allowed the attribution of the broad EPR line to the spin interaction between delocalized electrons in the graphene layers and localized *π*-electrons trapped in the extended aromatic structure, while the narrow line was associated with the mobile electrons trapped in small separated surface areas [30,40,44,45,46,47]. Earlier we proposed that these two lines can be related to two different types of carbon hybridizations, i.e., the broad line corresponds to *sp*^2^-hybridized carbon atoms, and the narrow line can be associated with *sp*^3^-hybridized and oxygen-bonded edge carbon atoms [24].

After the thermal treatment of CNFs_ox, both lines in the EPR spectrum broadened (Figure 9b). Broadening is usually associated with increasing spin relaxation time [26]. The width of the EPR line depends on magnetic dipole interactions, exchange power, local fields and heat motion [48], and it is very sensitive both to the oxygen content in the sample [24,46] and the content and nature of the adsorbed molecules [24]. The number of unpaired electrons of both the *N_n_* and *N_b_* types also changed: the *N_n_* value decreased, and *N_b_* value increased, although the sum (*N_n_* + *N_b_*) remained unchanged (Figure 9c). This fact can be explained by the changes in the CNF structure. The CNF surface was thermally defunctionalized, resulting in the disappearance of oxygen-containing groups and a decrease in the number of *sp^3^*-hybridized edge carbon atoms (Figure 8a) and the number of surface *N_n_* electrons. At the same time, the defectiveness of CNFs_ox_400 increased significantly, which led to an increase in *N_b_*. The concentration of unpaired surface and bulk electrons in the CNFs_ox and CNFs_ox_400 can be estimated as

(2) $$c=\frac{N\cdot s_{particle}}{s_{BET}}$$

where *N* is expressed in spin·g^−1^ (Table 1), and *S_particle_* is the area of a CNF particle (~450 nm^2^) assuming square particle with the side of 15 nm (Figure 1b). This quantitative evaluation shows that most of the electrons are located on the surface and are associated with edge and surface carbon atoms (Table 2).

### 4.2. Type of Ln^3+^ (Ln = La, Nd or Gd)

Paramagnetic ions affect nuclear spin–lattice relaxation both in solids and solutions [49]. The EPR spectra of La^3+^ are silent because of the absence of unpaired electrons in its electronic structure *[Xe]6s^0^5d^0^*. The EPR signal of Nd^3+^, which has a *[Xe]4f^3^* electronic structure, can be registered only at very low temperatures [50,51]. Gd^3+^ ions have a very large number of unpaired electrons due to their *[Xe]4f^7^* electronic structure. The best Gd^3+^ EPR spectra can be recorded in case of its very low (0.001–0.1 wt. %) content in the sample [52]. Otherwise, the dipole–dipole interaction blurs the energy levels and broadens the spectrum.

In this study, all Ln elements (La, Nd and Gd) had an effect on the EPR spectra of the composites. The Ln_2_O_3_/CNF EPR spectra are characterized by two *g*-factors in the range of 2.0011–2.0034 (Table 1). This means that oxide–carbon composites have two paramagnetic centers. The *g*-factors of Ln_2_O_3_/CNFs are lower than those of CNFs_ox and CNFs_ox_400 (Figure 9a).

The widths (Δ*H_n_*) of CNFs_ox and CNFs_ox_400 significantly exceed those of the Ln_2_O_3_/CNF composites. The EPR signal intensity is known to decrease in the order La(OH)_3_ > Nd(OH)_3_ > Gd(OH)_3_ because of a decrease in the number of oxygen vacancies [31]. In the present study, the Δ*H_b_* and Δ*H_n_* of Ln_2_O_3_/CNFs have a similar trend (La > Nd > Gd, Figure 9b) probably due to the same reason. However, we assume that the EPR spectra are mostly determined by the paramagnetic response of the carbon matrix.

The original hypothesis that metal ions (M^n+^) are coordinated on the surface of CNMs by purely Coulombic forces was later supplanted on the basis of calculation methods showing that *3d* and *4f* metal ions form chelated complexes on the surface of carbon particles [19,25,53]. Metal ions and molecules covalently bonded to the CNM surface affect the electronic structure of GO [54,55]. For example, the tyrazine molecules linked to the GO surface increase the number of unpaired electrons [56], while phosphorous atoms embedded into the graphene plane decrease the number of unpaired electrons [15]. The chemical bonding between Gd^3+^ ions and GO [26] or NDs [19,25] results in a decrease in free radical numbers and EPR signal intensity. In the present study, the number of unpaired electrons decreased by an order of magnitude for the Ln_2_O_3_/CNF samples compared to CNFs_ox and CNFs_ox_400 (Table 1). However, the fact that the La_2_O_3_/CNF composites had the maximum number of spins (Figure 9c) can be attributed to the least effective interaction between La^3+^ and the graphite support. The decrease in the Ln radii of La^3+^, Nd^3+^ and Gd^3+^ was respectively 1.03, 0.98 and 0.94 Å, and this resulted in an increase in energy of the Ln^3+^–graphene interaction [15]. The energy interaction correlated with ion type in the Ln_2_O_3_/CNF: the content of *sp*^3^-hybridized C (Figure 8a) and O atoms decreased in the La^3+^ > Nd^3+^ > Gd^3+^ series (Figure 8b).

### 4.3. Effect of Gd_2_O_3_ Weight Content on the Electronic Structure of the Gd_2_O_3_/CNF Composites

Gd^3+^ ions produce their own EPR spectrum, so it can be assumed that changes in their content in the Gd_2_O_3_/CNF composite will also affect the spectrum. Previously, it was found that an increase in Gd^3+^ content in the Gd^3+^/GO composite up to 15% resulted in the disappearance of the EPR signal. The authors attributed this fact to the disappearance of free radicals brought about by the complete bonding of Gd^3+^ with the GO surface [26]. In this study, the EPR spectra of all the composites with a Gd_2_O_3_ content of 20–50 wt. % demonstrated two paramagnetic responses (Figure S3). The *g*-factors (Figure 9a) and Δ*H* (Figure 9b) changed, while the number of unpaired spins *N_b_*, *N_n_* and their sum *N_b_* + *N_n_* (Figure 9c) decreased with an increase in the Gd^3+^ content of the sample. The increase in Gd_2_O_3_ content led to (1) a decrease in the carbon-related component and (2) an increase in the number of Gd^3+^ ions directly coordinated to the CNF surface. Gd^3+^ ions compensate surface carbon charge, which agrees with the results previously reported by Panich et al., who proposed that metal ions (M^n+^) interact with carbon-inherited electrons and with the oxygen of the functional groups [19,57].

## 5. Conclusions

We synthesized several types of surface-oxidized carbon nanoflakes decorated with Ln_2_O_3_ (Ln = La, Nd or Gd). The content of Ln_2_O_3_ in the composition of Ln_2_O_3_/CNFs varied from 20 up to 50 wt. %. The high content of Ln_2_O_3_ makes these particles potentially suitable for practical use as contrast agents for computed tomography and MRI. The problem of the stabilization of a large number of Ln^3+^ ions on the surface of carbon nanomaterials has been solved. Ln^3+^ ions were uniformly deposited on the carbon surface during the stage of composite synthesis because of C–O–Ln bond formation. These bonds remained after annealing, resulting in insoluble Ln_2_O_3_ and Ln_2_O_2_CO_3_ phases stabilized with CNFs. According to the EPR data, all composites were characterized by two types of paramagnetic centers despite the changes in the Ln^3+^ ion type and the changes in Gd content. However, the distribution of free surface electrons varied greatly. A correlation has been established between the number of *sp*^3^-hybridized edge carbon atoms and the number of unpaired electrons.

## Figures and Tables

**Table 1:** Linewidths (Δ*H*), *g*-factors and spin numbers (*N*) calculated from EPR spectra of samples. Subscripts *n* and *b* denote the narrow and broad components. Uncertainty regarding spin numbers is less than 15%.

Sample	Linewidth, G		*g*-Factor		Spin Number (*N*) (10^16^, Spin·g^−1^)	
	Δ*H_n_*	Δ*H_b_*	*g_n_*	*g_b_*	*N_n_*	*N_b_*
CNFs_ox	2.928	25.524	2.0020	2.0015	227.5	748.8
CNFs_ox_400	5.61	36.74	2.0037	2.0029	71.1	882.9
20La_2_O_3_/CNFs	7.908	34.6	2.0022	2.0019	129.2	633.7
20Nd_2_O_3_/CNFs	4.653	22.97	2.0017	2.0010	4.67	21.9
20Gd_2_O_3_/CNFs	4.501	21.39	2.0018	2.0015	12.3	50.7
30Gd_2_O_3_/CNFs	3.9	15.19	2.0016	2.0011	5.28	17.3
40Gd_2_O_3_/CNFs	3.91	15.84	2.0017	2.0013	4.03	13.3
50Gd_2_O_3_/CNFs	4.4	17.96	2.0033	2.0029	4.44	13.1

**Table 2:** The concentrations (*c*) of unpaired surface and bulk electrons in the CNFs_ox and CNFs_ox_400.

Sample	*S_BET_*, m^2^·g^−1^	*c_b_*, Spin («Bulk» Electrons)	*c_n_*, Spin («Surface» Electrons)
CNFs_ox	401	2.5	8.4
CNFs_ox_400	374	0.9	10.6

## Data Availability

The original contributions presented in this study are included in the article/Supplementary Material. Further inquiries can be directed to the corresponding authors.

## Supplementary Materials

The following supporting information can be downloaded at https://www.mdpi.com/article/10.3390/nano15131016/s1: Figure S1: HAADF-STEM images and EELS spectra of Ln_2_O_3_/CNFs, Ln = La (a), Nd (b) or Gd (c); Figure S2: C1s (a) and O1s (b) XPS deconvoluted spectra of Ln_2_O_3_/CNFs (Ln = La, Nd or Gd) with Ln_2_O_3_ weight content of 20, 30, 40 and 50 wt. %.; Figure S3: EPR (left) and absorption EPR (right) spectra of xLn_2_O_3_/CNFs (Ln = La (a), Nd (b) or Gd (c); x = 30 (d), 40 (e) or 50 (f)).
