# Supplementary material for: Influence of Content and Type of Lanthanide on the Structure of Ln2O3-Covered Carbon Nanoflakes: The EPR and XPS Study
Source: Nanomaterials (Basel). 2025 Jul 1;15(13):1016. doi: 10.3390/nano15131016 (PMC12250894; doi:10.3390/nano15131016)
Supplement: Supplementary file 1 [file nanomaterials-15-01016-s001.zip › nanomaterials-3646955-supplementary.pdf]

nanomaterials

Article

Supplementary Materials

Influence of Content and Type of Lanthanide on the Structure of Ln_2O_3-Covered Carbon Nanoflakes: The EPR and XPS Study

Serguei V. Savilov [1,*], Evgeniya V. Suslova [1,*], Alexander N. Ulyanov [1], Konstantin I. Maslakov [1], Sergey V. Maximov [1], Denis A. Shashurin [1,2] and Georgy A. Chelkov [1,3]

[1] Chemistry Department, Lomonosov Moscow State University, Moscow 119991, Russia; a-ulyanov52@yandex.ru (A.N.U.); nonvitas@gmail.com (K.I.M.); irber@yandex.ru (S.V.M.); shashurin@mail.ru (D.A.S.); chelkov@jinr.ru (G.A.C.)
[2] Faculty of Medicine, Medical Scientific-Educational Institute, Lomonosov Moscow State University, Moscow 119991, Russia
[3] Joint Institute for Nuclear Research, Dubna 141980, Russia
* Correspondence: savilov@chem.msu.ru (S.V.S.); suslova@kge.msu.ru (E.V.S.)

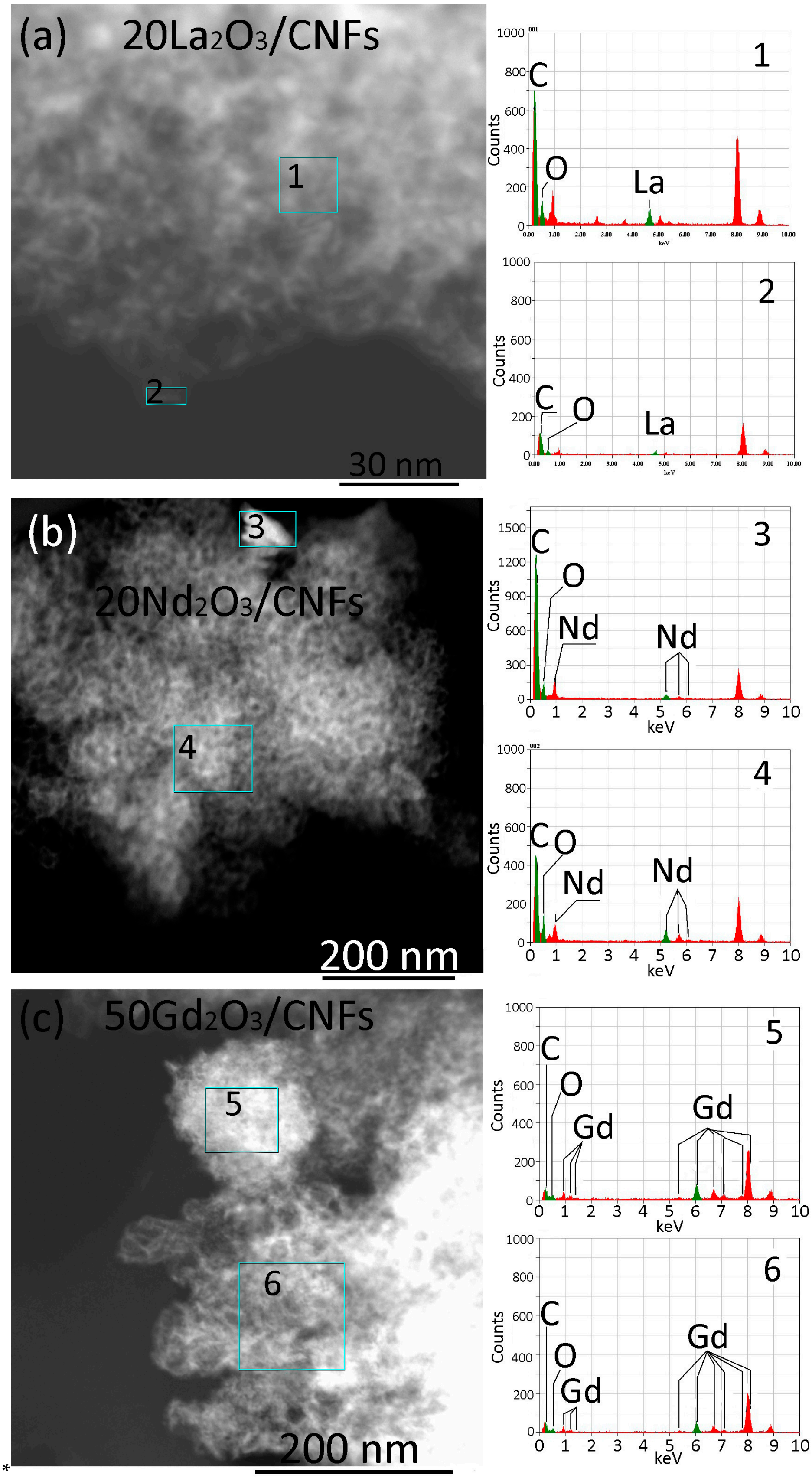

Figure S1. HAADF-STEM images and EELS spectra of Ln_2O_3/CNFs, Ln = La (a), Nd (b), Gd (c).

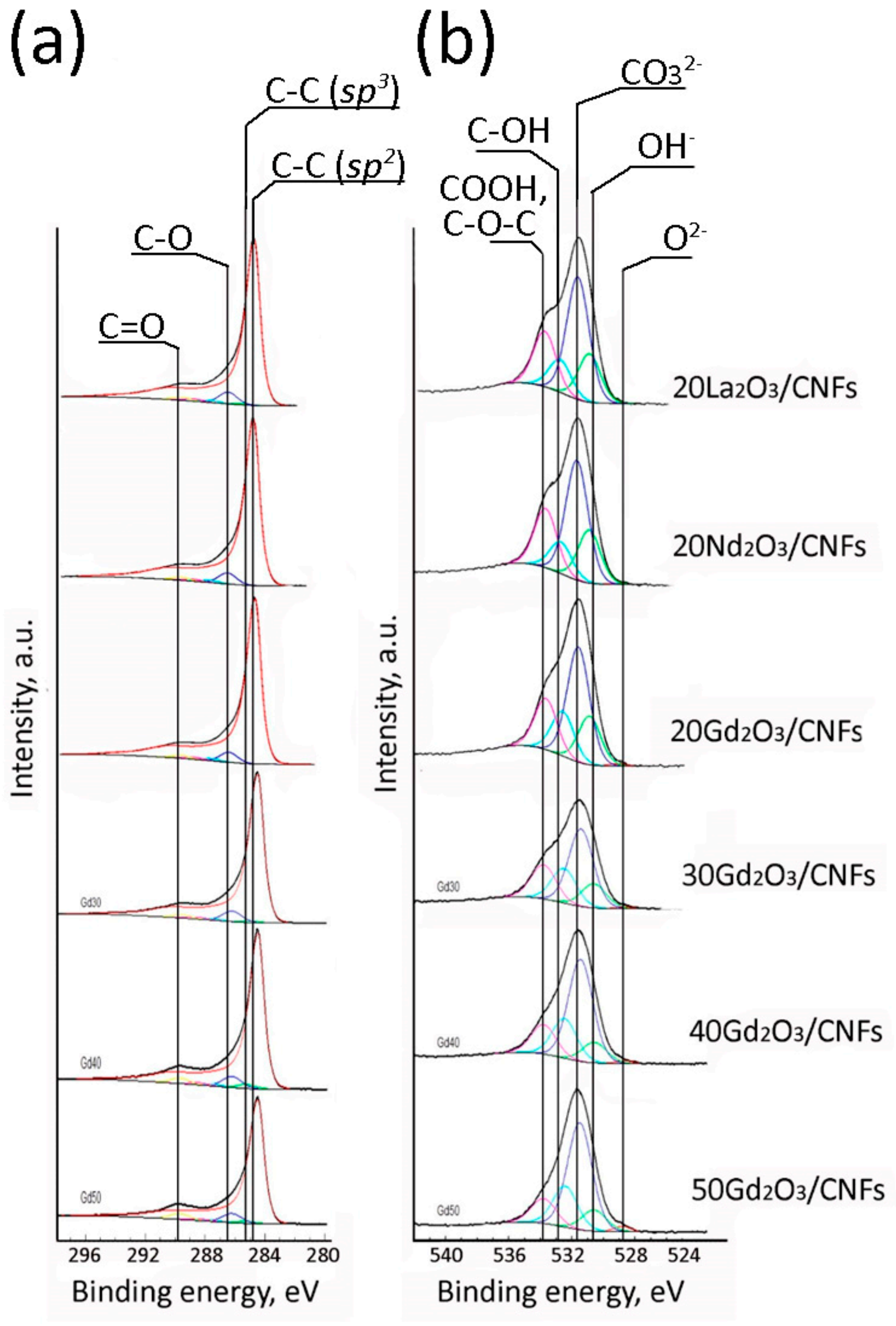

Figure S2. Curve-fitted C1s (a) and O1s (b) XPS spectra of Ln_2O_3/CNFs (Ln = La, Nd, Gd) with Ln_2O_3 weight contents of 20, 30, 40 and 50 wt. %.

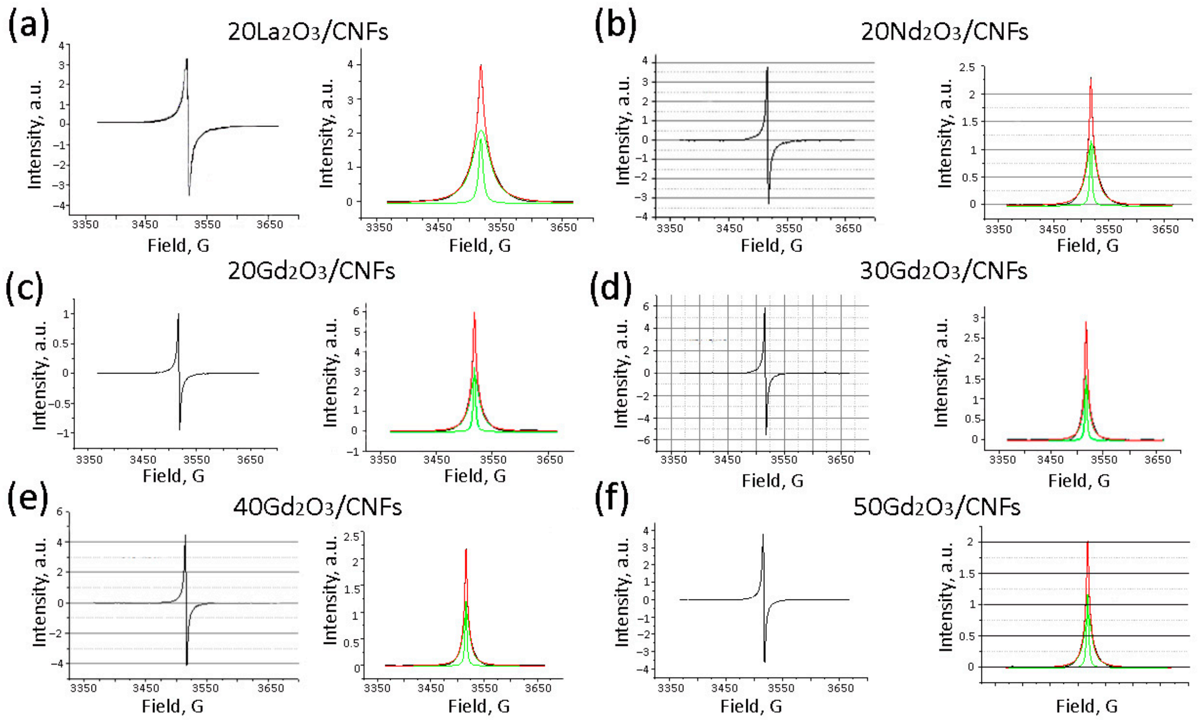

Figure S3. EPR (left) and absorption EPR (right) spectra of xLn_2O_3/CNFs (Ln = La (a), Nd (b), Gd (c); x = 30 (d), 40 (e), 50 (f)).
